# Sphingosine‐1‐phosphate acts as a key molecule in the direct mediation of renal fibrosis

**DOI:** 10.1002/phy2.172

**Published:** 2013-12-05

**Authors:** Shunji Shiohira, Takumi Yoshida, Hidekazu Sugiura, Miki Nishida, Kosaku Nitta, Ken Tsuchiya

**Affiliations:** 1 Department of Medicine IV, Tokyo Women's Medical University, Shinjuku, Tokyo, Japan; 2 Yoshida Medical Clinic, Suginami, Tokyo, Japan

**Keywords:** DMS, FTY720, renal fibrosis, sphingosine‐1‐phosphate, UUO

## Abstract

The major sphingolipid metabolite, sphingosine‐1‐phosphate (S1P), has important biological functions. S1P serves as a ligand for a family of five G‐protein‐coupled receptors with distinct signaling pathways regulating important biological pathways. S1P induces renal fibrosis through an inflammatory pathway. However, its direct fibrosis‐inducing effect on the kidney has not been shown. The role of S1P as a direct mediator of renal fibrosis was investigated in normal rat kidney interstitial fibroblast (NRK‐49F) cells (in vitro) and kidneys of a unilateral ureteral obstruction (UUO) mouse model (in vivo). To clarify the role of S1P in renal fibrosis, we adopted nude UUO mice with immune response deficits. NRK‐49F cells were stimulated with various concentrations of exogenous S1P and FTY720 (a S1P receptor agonist) or *N*,*N*‐dimethylsphingosine (DMS; a sphingosine kinase inhibitor). C57BL6 and nude UUO mice were pretreated with FTY720, DMS, or saline. Expression levels of alpha‐smooth muscle actin (a‐SMA), E‐cadherin, collagen type 1 (COL1), collagen type 4 (COL4), tissue inhibitor of matrix metalloproteinase‐1 (TIMP1), and plasminogen activator inhibitor‐1 (PAI1) were examined. S1P stimulated fibrosis in NRK‐49F cells and UUO mice. Increased a‐SMA, COL1, COL4, TIMP1, and PAI1 and decreased E‐cadherin expression levels were observed in both the S1P‐stimulated cells and UUO mice. Nude UUO mouse kidneys expressed fibrotic markers. Fibrotic changes were successfully induced in both UUO and nude UUO mice, evident through prominent fibronectin and COL1 staining. These S1P‐induced fibrotic changes were suppressed by FTY720 and DMS both in vitro and in vivo. Thus, S1P essentially and directly mediates renal fibrosis.

## Abstract (precis)

Sphingosine‐1‐phosphate (S1P) stimulated fibrosis both in vitro and in vivo. Fibrotic changes were successfully induced in both unilateral ureteral obstruction (UUO) and nude mice with UUO. These results suggest that S1P is a pivotal fibrotic mediator in renal fibrosis that acts, in part, through direct fibrotic effects.

## Introduction

Renal fibrosis is the final common manifestation of a wide variety of chronic kidney diseases (CKDs) (Hodgkins and Schnaper 2012). Pathological findings of renal fibrosis include glomerulosclerosis, tubulointerstitial fibrosis, inflammatory cell infiltration, and loss of renal parenchyma characterized by tubular atrophy, capillary loss, and podocyte depletion (Lee and Kalluri 2010). The underlying cellular events that lead to these histological presentations are complicated and include mesangial and fibroblast activation, tubular epithelial to mesenchymal transition, monocyte/macrophage and T‐cell infiltration, and cellular apoptosis (Barnes and Glass 2011). Transforming growth factor‐beta (TGF‐*β*) and connective tissue growth factor (CTGF) are important factors in the pathogenesis of fibrosis (Wang et al. 2011).

Sphingolipid metabolites are emerging as important lipid signaling molecules in both health and disease (Arana et al. 2010). Among them, sphingosine‐1‐phosphate (S1P), produced by phosphorylation of sphingosine by sphingosine kinases in response to various stimuli, plays important roles in a number of cellular processes, including cell growth and cell trafficking (Salata et al. 2012).

Tissue distribution of the S1P receptor (S1PR) subtypes and the differing signaling pathways and downstream cellular effects resulting from their activation underscore the need for the discovery and testing of novel subtype‐specific S1PR compounds for the treatment of various disorders (Huwiler and Pfeilschifter 2008). Several reports have suggested that the fibrotic effects of TGF‐*β* may be partially mediated through the S1P pathway (Watterson et al. 2007) and that S1P stimulates the expression of CTGF, a protein implicated in numerous fibrotic disorders (Hla et al. 2012).

Although there have been reports of S1P inducing fibrotic changes in other tissues, there have been no reports of direct fibrotic effects of S1P on the kidney. Here, the role of S1P as a mediator of renal fibrosis was investigated in normal rat kidney interstitial fibroblast (NRK‐49F) cells and in the kidneys of a mouse model of unilateral ureteral obstruction (UUO). To clarify the role of S1P in renal fibrosis, we adopted a UUO model in nude mice, which are characterized by immune response deficits.

## Material and Methods

### Experimental protocol (in vitro)

NRK‐49F cells were stimulated with exogenous S1P (0.1, 1.0, or 2.0 *μ*mol/L) (Cayman Chemical Company, Ann Arbor, MI), and the mRNA and protein expression levels of alpha‐smooth muscle actin (a‐SMA), E‐cadherin, collagen type 1 (COL1), collagen type 4 (COL4), tissue inhibitor of matrix metalloproteinase‐1 (TIMP1), and plasminogen activator inhibitor‐1 (PAI1) were examined. Morphological changes and migration of the NRK‐49F cells after stimulation by S1P (0.5, 1.0, or 2.0 *μ*mol/L) for 1 day were examined. Fibrotic changes and migration of the NRK‐49F cells induced by S1P were then evaluated after the addition of FTY720 (0.5, 1.0, or 2.0 *μ*mol/L) (Cayman Chemical Company) or *N*,*N*‐dimethylsphingosine (DMS; 0.5, 1.0, or 2.0 *μ*mol/L) (Cayman Chemical Company) for 1 day. The prototype S1PR modulator, FTY720, has been shown to target four of the five S1PR subtypes (1, 3, 4, and 5) and to act at several levels to modulate lymphocyte trafficking through lymphocytic and endothelial S1P1 and perhaps through other inflammatory processes with additional S1PR subtypes (Roberts et al. 2013). DMS, a sphingosine kinase inhibitor, has been shown to inhibit sphingosine kinase activity in several cell types (McDonald et al. 2010).

### Experimental animal model

The animals were given free access to standard food and water and were cared for in accordance with the Institutional Animal Research Committee's Guide for the Care and Use of Laboratory Animals, published by the U.S. National Institutes of Health (NIH publication number 85–23, revised 1996). Our animal experiment protocols were approved by an Independent Ethics Committee of Tokyo Women's Medical University. All of the animals were anesthetized with pentobarbital (40–50 mg/kg) prior to surgery.

Renal fibrosis was induced by a UUO operation, as described previously (Sugiura et al. 2012), in male C57BL6 mice and congenitally athymic BALB/c AJcl‐nu/nu (nude) mice (CLEA Japan Inc., Tokyo, Japan) at 6 weeks of age. The C57BL6 mice and nude UUO mice were pretreated with FTY720 (1 mg/kg/day), DMS (1 mg/kg/day), or saline using an osmotic pump (Alzet, micro‐osmotic pump, #1007D; Cupertino, CA) for 3 or 7 days. These mice were sacrificed 3 or 7 days after UUO in order to harvest the kidneys for RNA extraction and immunohistochemistry. The mRNA and/or protein expression levels of a‐SMA, E‐cadherin, COL1, COL4, TIMP1, and PAI1 were examined. The samples were subjected to immunostaining (fibronectin and COL1) and immunofluorescence staining (a‐SMA and fibronectin).

### Cell culture

NRK‐49F cells, which represent normal rat kidney fibroblast cells, were grown in 4 mL of medium (minimum essential medium; Life Technologies Corporation, Tokyo, Japan) at 98.6 Fahrenheit. The medium was supplemented with 10% fetal bovine serum (FBS) (Life Technologies Corporation), 100 units/mL penicillin, 100 *μ*g/mL streptomycin (Life Technologies Corporation), and minimum essential medium nonessential amino acid solution, 100× (11140‐050). The medium was changed every 1–2 days until the cultures became confluent (90%), and the cultures were maintained for 2 days in 6‐well dishes with membrane filter medium containing 1% FBS.

### RNA isolation and real‐time polymerase chain reaction

Expression levels of fibrotic mediators were examined by real‐time polymerase chain reaction (RT‐PCR). RNA isolation and RT‐PCR were performed as described previously (Yoshida et al. 2008). In brief, total RNA was extracted from the cells and kidneys with TRIzol Reagent (Life Technologies Corporation). cDNA was prepared using a High Capacity cDNA Reverse Transcription kit (Applied Biosystems, Tokyo, Japan) for analysis by RT‐PCR with Step One™(Applied Biosystems), Power SYBR Green^®^ PCR Master Mix, and specific primers. The mRNA levels were normalized to the primer for rodent glyceraldehyde‐3‐phosphate dehydrogenase (GAPDH). The specific primers used for the fibrotic mediators were as follows:

a‐SMA: CTCTGGTGTGTGACAATGGTCC (forward) and CGAAGCTCGTTATAGAAGGAGTG (reverse), E‐cadherin: GCAGTTCTGCCAGAGAAACC (forward) and TGGATCCAAGATGGTGATGA (reverse), COL1: TCGAGTATGGAAGCGAAGGT (forward) and TTGAGGTTGCCAGTCTGTTG (reverse), COL4: CCACACTATCCGCAGACTGA (forward) and TGTGTGTGCTCAGGTGTGAA (reverse), PAI‐1: GCAACAAGAGCCAATCACAA (forward) and ACATCTGCATCCTGGAGCTT (reverse), and TIMP1: TCCCTTGCAAACTGGAGAGT (forward) and TATTGCCAGGTGCACAAATC (reverse).

### Antibodies and reagents

Monoclonal anti‐a‐SMA was purchased from Sigma‐Aldrich Co. LLC (Tokyo, Japan). E‐cadherin, COL1, and PAI1 were purchased from Santa Cruz Biotechnology Inc (Dallas, TX). COL4 and TIMP1 were purchased from Bioworld Technology Inc (St. Louis Park, MN). Fibronectin was purchased from BD Biosciences (Tokyo, Japan) and Sigma‐Aldrich Co. LLC. GAPDH was purchased from Novus Biologicals LLC (Littleton, CO).

### Western blotting

The cells were harvested and lysed in a buffer containing 20 mmol/L 1‐Piperazineethanesulfonic acid, 4‐(2‐hydroxyethyl)‐, monosodium salt (Na‐HEPES) (pH 7.5), 100 mmol/L NaCl, 1% Triton X‐100, 15 mmol/L NaF, 1 mmol/L Na_3_VO_4_, 10 mmol/L Na_4_P_2_O_7_, 1 mmol/L ethylenediaminetetraacetic acid (EDTA), protease inhibitor cocktail (Sigma‐Aldrich Co. LLC), and phosphatase inhibitor cocktail (Roche Diagnostics Corporation, Indianapolis, IN). Frozen kidney samples were homogenized on ice in lysis buffer and centrifuged at 10,000 rpm for 10 min to remove debris. Laemmli sample buffer (Bio‐Rad Laboratories Inc., Tokyo, Japan) was added to the samples and denatured at 96°C for 5 min. The samples were then subjected to sodium dodecyl sulfate polyacrylamide gel electrophoresis (SDS‐PAGE). Ten microgram of protein from each specimen was separated on a 4–15% PAGE (Criterion TGX Precast gels; Bio‐Rad Laboratories Inc.) and electrophoretically transferred to a nitrocellulose membrane. The membrane was blocked with 5% skim milk in 0.1% Tween 20 in Tris‐buffered saline (TBS‐T). Antibodies for a‐SMA, E‐cadherin, fibronectin, TIMP1, PAI‐1, and GAPDH were used for immunoblotting. The membranes were incubated with primary antibodies overnight at 4°C. After three separate 5‐min washes with TBS‐T, the membranes were incubated for 1 h at room temperature with horseradish peroxidase‐conjugated secondary antibodies (anti‐mouse for a‐SMA, PAI1, and GAPDH; anti‐rabbit for E‐cadherin; and anti‐chicken for fibronectin). After three separate 10‐min washes, the membranes were incubated with an ECL western blotting system (GE Healthcare, Tokyo, Japan). Bands were visualized by the enhanced chemiluminescence method, and their intensities were quantified using Multi Gauge software (Fujifilm Holdings Corporation, Tokyo, Japan).

### Histologic evaluations

For evaluate interstitial fibrosis stained with hematoxylin and eosin (magnification of 200×), the scoring systems were quoted from a previous report (Depierreux et al. 1994). The definitions were as follows: tubular atrophy: 0, normal tubules; 1, rare single atrophic tubule; 2, several clusters of atrophic tubules; 3, massive atrophy; tubular necrosis: 0, normal tubules; 1, rare single necrotic tubule; 2, several clusters of necrotic tubules; 3, massive necrosis; lymphocytic infiltrates: 0, absent; 1, few scattered cells; 2, groups of lymphocytes; 3, widespread infiltrate; interstitial fibrosis: 0, absent; 1, minimal fibrosis, with slight thickening of the tubular basal membrane; 2, moderate fibrosis, with focal enlargement of the interstitium; 3, severe fibrosis, with confluent fibrotic areas. Tubulointerstitial injury scores were defined as the sum of the four scores.

### Immunofluorescence staining

Cells were incubated on cover glass. The cells were fixed with 4% paraformaldehyde in phosphate‐buffered saline (PBS) for 30 min at room temperature, and permeabilization was performed with methanol for 20 min at ‐20°C. After the cells were blocked with blocking buffer (5% goat serum in PBS) for 30 min at room temperature, the cells were incubated in a primary antibody solution overnight at 4°C. The cells were then incubated in a secondary antibody solution for 40 min at room temperature in the dark and exposed to 4′,6‐diamidino‐2‐phenylindole dye for 4 min. Finally, the cells were mounted with Vectashield (Vector Laboratories Inc., Burlingame, CA).

For immunofluorescence staining, primary antibodies against a‐SMA and fibronectin were used at 1:100 dilution, and secondary antibodies were used at 1:100 dilution.

### Immunohistochemical staining

The kidneys were fixed with 4% paraformaldehyde in PBS for 16 h at 4°C and then embedded in paraffin. Before immunohistochemical staining, paraffin sections (3 *μ*m thick) were deparaffinized and hydrated, and antigen retrieval was performed in a microwave oven in 0.1 mol/L Citrate buffer, pH 6.0, for 30 min. Endogenous tissue peroxidase activity was blocked by incubation in 0.3% H_2_O_2_ in methanol for 30 min. A Vectastain Elite ABC standard kit (Vector Laboratories Inc.) was used. For fibronectin, a Vector M.O.M.™ Immunodetection kit (Funakoshi Corporation, Tokyo, Japan) was used. The sections were incubated with blocking buffer (COL1A1, 5% rabbit serum in TBS) for 1 h and the primary antibodies against fibronectin (dilution 1:50) or COL1 (dilution 1:200) overnight at 4°C. After incubation with the appropriate biotinylated secondary antibodies for 1 h, the sections were incubated with Vectastain ABC reagent for 30 min and stained using a peroxidase substrate solution DAB substrate kit (Vector Laboratories Inc.). The results were quantified by measuring the relative densities using the ImageJ software program, which is an open source Java program created by NIH; it is used for many imaging applications (http://rsbweb.nih.gov/ij/). The results were expressed as the ratio of the positive area to the total scanned interstitium area in five randomly selected fields.

### Scratch wound‐healing assay

The effects on migration were investigated using Biostation CT (Nikon Instruments, Tokyo, Japan). When the cultures were at ~90% confluence, linear wounds were drawn across the center of each well by cell scraping. Scratch wound healing was examined using a microscope (Nikon BioStation CT) 48 h after stimulation by S1P. FTY720 (100 or 200 ng/mL) or DMS (250 or 500 ng/mL) was added. After 1 h, 0.1 *μ*mol/L of S1P was added, and the cells were stimulated.

### Statistical analyses

JMP version 10 (SAS Institute Inc., Tokyo, Japan) was used to analyze and present the data. All of the data are reported as means ± standard error (SE). Differences between the groups were analyzed by Student's *t*‐tests or one‐way analyses of variance (ANOVA). *P* < 0.05 was considered to indicate significance.

## Results

### Effects of S1P on expression levels of fibrotic mediators in NRK‐49F cells (*RT*‐PCR)

**Figure 1.**
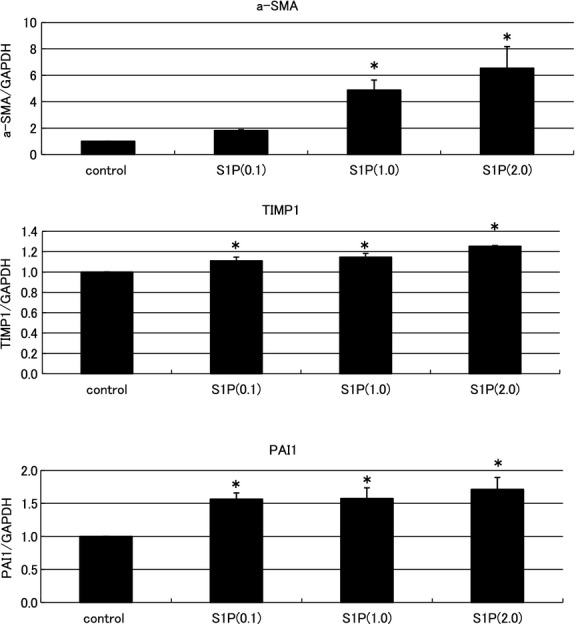
Examination of the effects of sphingosine‐1‐phosphate (S1P) on the expression levels of fibrotic mediators in normal NRK‐49F cells by real‐time PCR. S1P treatment stimulated the expression of fibrotic mediators in the NRK‐49F cells. Significantly increased expression levels of alpha‐smooth muscle actin (a‐SMA), tissue inhibitor of matrix metalloproteinase‐1 (TIMP1), and plasminogen activator inhibitor‐1 (PAI1) were observed in the S1P‐stimulated cells. The increases exhibited a dose‐dependent pattern. *N* = 5. Data are presented as means ± standard error (SE). *P < 0.05. NRK‐49F, normal rat kidney interstitial fibroblast.

At first, the role of S1P as a fibrotic mediator in the kidney was investigated in an in vitro study using renal interstitial fibroblast cells. NRK49F cells are suitable for the observation of renal fibrosis. S1P treatment stimulated the expression of fibrotic mediators in the NRK‐49F cells. The a‐SMA, TIMP1, and PAI1 expression levels increased significantly in the S1P‐stimulated cells (a‐SMA/GAPDH ratio: control to S1P 0.1 *μ*mol/L: from 1.0 ± 0.0 to 1.82 ± 0.05, S1P 1.0 *μ*mol/L: from 1.0 ± 0.0 to 4.88 ±0.76, S1P 2.0 *μ*mol/L: from 1.0 ± 0.0 to 6.54 ± 1.63; TIMP1/GAPDH ratio: control to S1P 0.1 *μ*mol/L: from 1.0 ± 0.0 to 1.11 ± 0.04, S1P 1.0 *μ*mol/L: from 1.0 ± 0.0 to 1.15 ± 0.04, S1P 2.0 *μ*mol/L: from 1.0 ± 0.0 to 1.25 ± 0.01; PAI‐1/GAPDH ratio: control to S1P 0.1 *μ*mol/L: from 1.0 ± 0.0 to 1.57 ± 0.10, S1P 1.0 *μ*mol/L: from 1.0 ± 0.0 to 1.57 ± 0.16, S1P 2.0 *μ*mol/L: from 1.0 ± 0.0 to 1.71 ± 0.18; Fig. 1). These results suggest that S1P is a fibrotic mediator in vitro.

### Antifibrotic effects of FTY720 and DMS in NRK‐49F cells (*RT‐*PCR)

**Figure 2.**
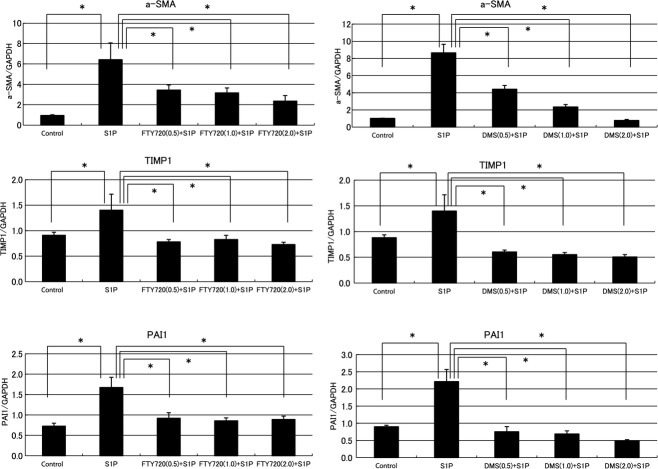
Examination of the antifibrotic effects of FTY720 and *N*,*N*‐dimethylsphingosine (DMS) in NRK‐49F cells by real‐time PCR. S1P treatment stimulated the expression of fibrotic mediators in the NRK‐49F cells. Significantly increased expression levels of a‐SMA, TIMP1, and PAI1 were observed in the S1P‐stimulated cells. These increases were suppressed in the presence of FTY20, and the antifibrotic effects of FTY720 exhibited a dose‐dependent pattern. In the presence of DMS, the cellular effects of S1P were attenuated in a manner similar to those with FTY720. These antifibrotic effects of DMS exhibited a dose‐dependent pattern. *N* = 8. Data are presented as means ± SE. *P < 0.05. S1P, sphingosine‐1‐phosphate; NRK‐49F, normal rat kidney interstitial fibroblast; a‐SMA, alpha‐smooth muscle actin; TIMP1, tissue inhibitor of matrix metalloproteinase‐1; PAI1, plasminogen activator inhibitor‐1.

Figure 1 shows that S1P is a fibrotic mediator in vitro. Next, the relationships between S1P and compound‐related phospholipids, such as FTY720 and DMS, were investigated in vitro. S1P treatment stimulated the expression of fibrotic mediators in NRK‐49F cells. The a‐SMA, TIMP1, and PAI1 expression levels increased significantly in the S1P‐stimulated cells. In the presence of FTY720, these S1P‐induced fibrotic changes in the NRK‐49F cells were suppressed significantly (a‐SMA/GAPDH ratio: control to FTY720 0.5 *μ*mol/L: from 6.42 ± 1.65 to 3.43 ± 0.52, FTY720 1.0 *μ*mol/L: from 6.42 ± 1.65 to 3.17 ± 0.48, FTY720 2.0 *μ*mol/L: from 6.42 ± 1.65 to 2.35 ± 0.55; TIMP1/GAPDH ratio: control to FTY720 0.5 *μ*mol/L: from 1.40 ± 0.31 to 0.78 ± 0.05, FTY720 1.0 *μ*mol/L: from 1.40 ± 0.31 to 0.83 ± 0.08, FTY720 2.0 *μ*mol/L: from 1.40 ± 0.31 to 0.73 ± 0.04; PAI‐1/GAPDH ratio: control to FTY720 0.5 *μ*mol/L: from 1.67 ± 0.25 to 0.92 ± 0.13, FTY720 1.0 *μ*mol/L: from 1.67 ± 0.25 to 0.86 ± 0.07, FTY720 2.0 *μ*mol/L: from 1.67 ± 0.25 to 0.89 ± 0.08; Fig. 2). The antifibrotic effects of FTY720 exhibited a dose‐dependent pattern. In the presence of DMS, the cellular effects of S1P were attenuated similar to that observed in the presence of FTY720 (a‐SMA/GAPDH ratio: control to DMS 0.5 *μ*mol/L: from 8.66 ± 0.99 to 4.42 ± 0.43, DMS 1.0 *μ*mol/L: from 8.66 ± 0.99 to 2.36 ± 0.28, DMS 2.0 *μ*mol/L: from 8.66 ± 0.99 to 0.78 ± 0.11; TIMP1/GAPDH ratio: control to DMS 0.5 *μ*mol/L: from 1.40 ±0.31 to 0.61 ± 0.04, DMS 1.0 *μ*mol/L: from 1.40 ± 0.31 to 0.55 ± 0.04, DMS 2.0 *μ*mol/L: from 1.40 ± 0.31 to 0.51 ± 0.05; PAI‐1/GAPDH ratio: control to DMS 0.5 *μ*mol/L: from 2.21 ± 0.35 to 0.75 ± 0.15, DMS 1.0 *μ*mol/L: from 2.21 ± 0.35 to 0.69 ± 0.09, DMS 2.0 *μ*mol/L: from 2.21 ± 0.35 to 0.48 ± 0.04; Fig. 2).

### Changes in expression levels of SIP‐induced fibrotic mediators in NRK‐49F cells (western blotting)

**Figure 3.**
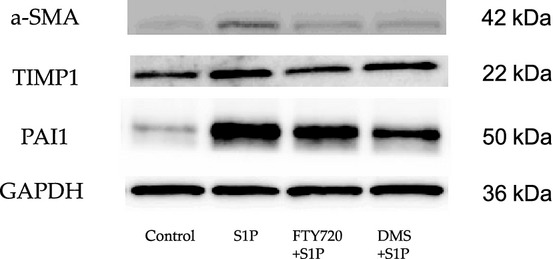
Examination of the changes in the expression levels of S1P‐induced fibrotic mediators by western blotting. The relationship between S1P and fibrosis was examined at the protein level. S1P induced fibrosis at the protein and genetic levels. S1P upregulated the protein expression levels of a‐SMA, TIMP1, and PAI‐1 in the NRK‐49F cells. A representative western blot is shown. These effects on the protein levels were attenuated by FTY720 and DMS addition. S1P, sphingosine‐1‐phosphate; a‐SMA, alpha‐smooth muscle actin; TIMP1, tissue inhibitor of matrix metalloproteinase‐1; PAI1, plasminogen activator inhibitor‐1; NRK‐49F, normal rat kidney interstitial fibroblast; DMS, *N*,*N*‐dimethylsphingosine.

In addition to that at the mRNA level, the relationship between S1P and fibrotic mediator was examined at the protein level. At the protein level, western blotting showed that S1P increased the a‐SMA, TIMP1, and PAI1 expression levels in NRK‐49F cells. A representative western blot is shown in Figure 3. These effects on the protein levels were attenuated by FTY720 and DMS addition. Thus, at the protein level, S1P induced fibrotic mediators and FTY720 and DMS inhibited fibrotic mediators in vitro.

### Immunofluorescence staining

**Figure 4.**
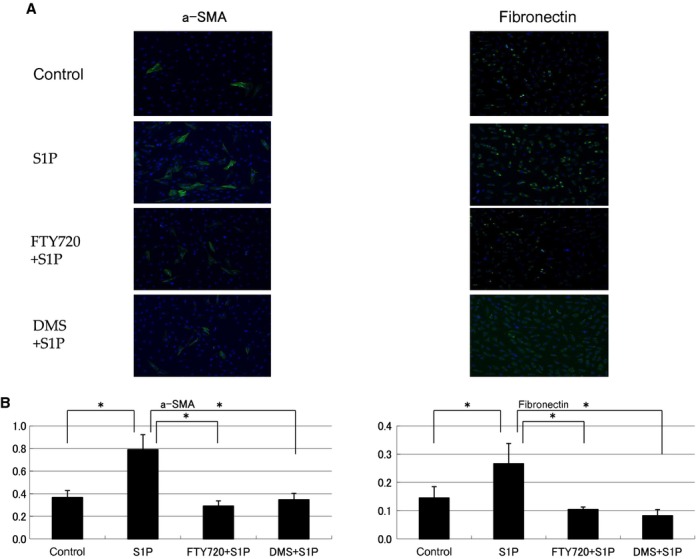
Examination of immunofluorescence staining and the percent area stained in NRK‐49F cells. NRK‐49F cells were stimulated by S1P, and after FTY720 or DMS addition, the cells were evaluated by immunofluorescence staining for a‐SMA and fibronectin (A). The percent area of the cells stained was evaluated (B). FTY720 and DMS pretreatment significantly inhibited S1P‐induced a‐SMA and fibronectin expression. Data are presented as means ± SE. *P < 0.05. NRK‐49F, normal rat kidney interstitial fibroblast; DMS, *N*,*N*‐dimethylsphingosine; a‐SMA, alpha‐smooth muscle actin; S1P, sphingosine‐1‐phosphate.

Immunofluorescence staining showed that S1P strongly participated in fibrosis. NRK‐49F cells were stimulated by S1P. After FTY720 or DMS addition, the cells were evaluated by immunofluorescence staining for a‐SMA and fibronectin (Fig. 4A). The percent area stained in the cells was also evaluated (Fig. 4B). FTY720 and DMS pretreatment significantly inhibited the expression levels of S1P‐induced a‐SMA and fibronectin (a‐SMA: control to S1P: from 0.37 ± 0.06 to 0.79 ± 0.13, S1P to FTY720: from 0.79 ± 0.13 to 0.29 ± 0.04, S1P to DMS: from 0.79 ± 0.13 to 0.35 ± 0.06; fibronectin: control to S1P: from 0.15**± 0.04 to 0.27**± 0.07, S1P to FTY720: from 0.27 ± 0.07 to 0.10 ± 0.01, S1P to DMS: from 0.27 ± 0.07 to 0.08 ± 0.02).

### Scratch wound‐healing assay in NRK‐49F cells

**Figure 5.**
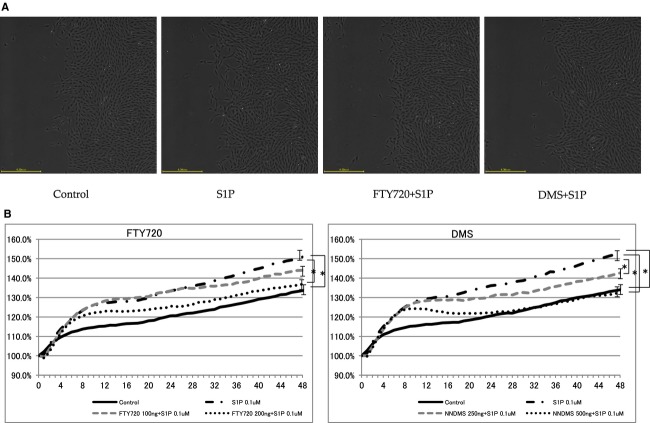
Scratch wound‐healing assay in NRK‐49F cells. S1P‐stimulated NRK‐49F cells exhibited significantly increased migration compared with that in the control. These effects were inhibited by FTY720 and DMS in a dose‐dependent manner. These bar graphs show mean. *N *=**8. Data are presented as means ± SE. *P < 0.05. NRK‐49F, normal rat kidney interstitial fibroblast; DMS, *N*,*N*‐dimethylsphingosine; S1P, sphingosine‐1‐phosphate.

Cell migration is an important factor in fibrosis. Thus, in this study, the relationship between S1P and fibrotic mediator was investigated by a cell migration assay. S1P‐stimulated NRK‐49F cells induced significantly more migration than controls (FTY720: control to S1P: from 127.10 ± 2.77% to 147.10 ± 3.19%; DMS: control to S1P: from 128.31 ± 2.25% to 156.17 ± 3.42%; Fig. 5). These effects were significantly inhibited by FTY720 and DMS (FTY720: S1P to FTY720 100**ng: from 147.10 ± 3.19% to 141.21 ± 2.83%, S1P to FTY720 200**ng: from 147.10 ± 3.19% to 134.80 ± 2.57%; DMS: S1P to DMS 250**ng: from 156.17 ± 3.42% to 144.84 ± 2.10%, S1P to DMS 500**ng: from 156.17 ± 3.42% to 137.08 ± 2.45%, DMS; Fig. 5). In this study, the changes in expression were examined at the gene and protein levels, and the morphology was investigated. These results also suggest that S1P was involved in the fibrotic changes observed in the kidney cells.

### Antifibrotic effects of FTY720 and DMS (UUO)

**Figure 6.**
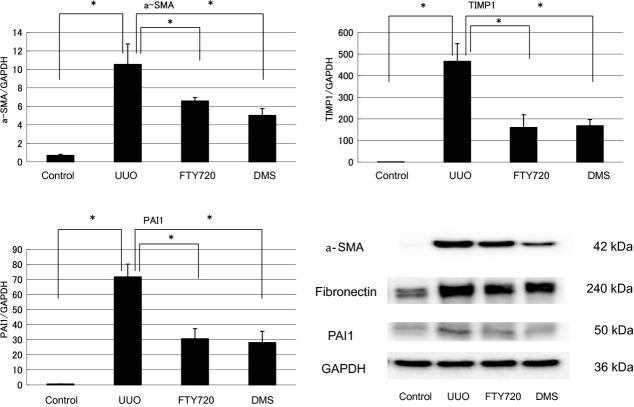
Examination of the antifibrotic effects of FTY720 and DMS in a UUO by real‐time PCR and western blotting. The role of S1P in fibrosis was examined in an in vivo model. In vivo, the mRNA expression levels of the various fibrotic markers were significantly increased in the left kidney compared with the right control kidney. FTY720 and DMS pretreatment significantly inhibited S1P‐induced fibrotic changes in the UUO model. (*N* = 5) UUO treatment with S1P upregulated the protein expression levels of a‐SMA, fibronectin, and PAI1. A representative western blot is shown. These effects on the protein levels were attenuated by FTY720 or DMS addition. Data are presented as means ± SE. *P < 0.05. NRK‐49F, normal rat kidney interstitial fibroblast; DMS, *N*,*N*‐dimethylsphingosine; S1P, sphingosine‐1‐phosphate; UUO, unilateral ureteral obstruction; a‐SMA, alpha‐smooth muscle actin; PAI1, plasminogen activator inhibitor‐1.

**Figure 7.**
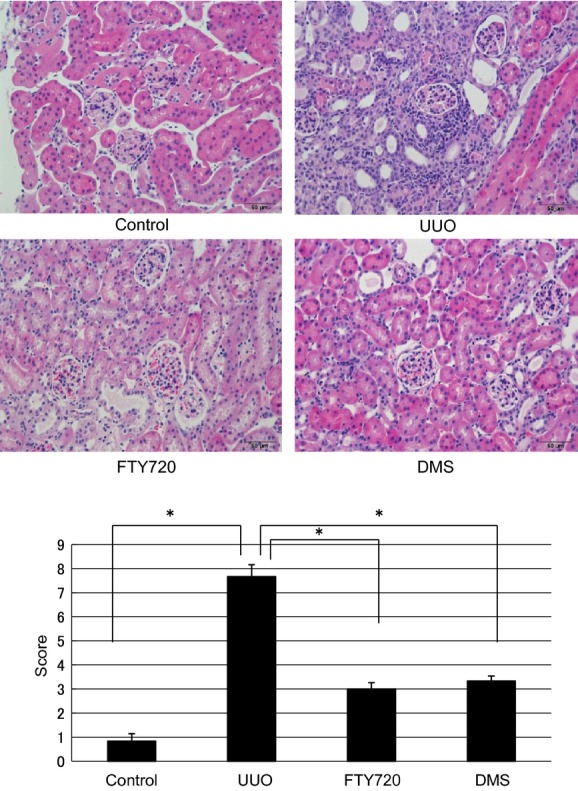
Examination of hematoxylin and eosin staining in the UUO model (magnification of 400×). In the interstitial injury score, the UUO mice exhibited increased the score in the left kidney compared with the control right kidney (from 0.83 ± 0.31 to 7.67 ± 0.49), and FTY720 and DMS pretreatment significantly decreased the scores (FTY720: from 7.67 ± 0.49 to 3.0 ± 0.26, DMS: from 7.67 ± 0.49 to 3.33 ± 0.21). *N* = 6. Data are presented as means ± SE. *P < 0.05. UUO, unilateral ureteral obstruction; DMS, *N*,*N*‐dimethylsphingosine.

**Figure 8.**
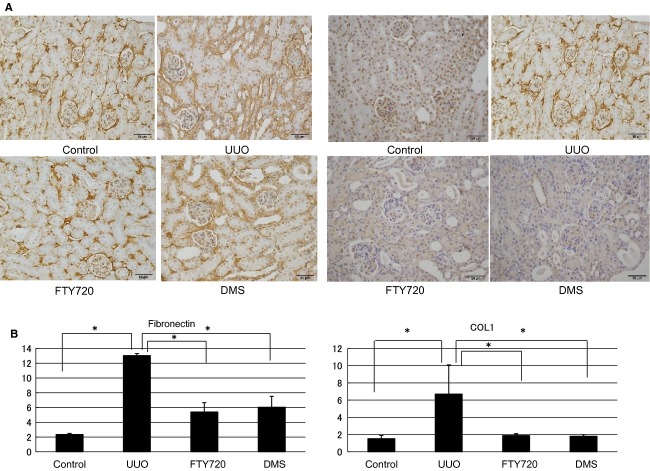
Examination of immunohistochemical staining in the UUO model. Immunohistochemistry showed that in the kidney of the UUO mouse, the stained area in the left kidney was increased compared with that in the control right kidney, and pretreatment with FTY720 and DMS significantly decreased the staining intensity of fibronectin and COL1. *N* = 3. Data are presented as means ± SE. *P < 0.05. UUO, unilateral ureteral obstruction; DMS, *N*,*N*‐dimethylsphingosine.

The findings of the participation of S1P in fibrosis were confirmed by in vivo examination. In the in vivo investigation, the mRNA expression levels of various fibrotic markers in the left kidney were significantly increased compared with those in the right kidney, which was used as a control (a‐SMA/GAPDH ratio: from 0.69 ± 0.12 to 10.53 ± 2.21, TIMP1/GAPDH ratio: from 0.81 ± 0.04 to 466.64 ± 81.81, PAI1/GAPDH ratio: from 0.53 ± 0.05 to 71.71 ± 8.46; Fig. 6). FTY720 or DMS pretreatment significantly inhibited the S1P‐induced fibrotic changes in UUO mice (a‐SMA/GAPDH ratio: after FTY720: from 10.53 ± 2.21 to 6.58 ± 0.37, after DMS: from 10.53 ± 2.21 to 5.02 ± 0.74; TIMP1/GAPDH ratio: after FTY720: from 466.64 ± 81.81 to 160.19 ±59.12, after DMS: from 466.64 ± 81.81 to 168.68 ±28.21; PAI‐1/GAPDH ratio: after FTY720: from 71.71 ± 8.46 to 30.59 ± 6.74, after DMS: from 71.71 ±8.46 to 28.09 ± 7.49; Fig. 6). Moreover, similar results were obtained in the western blot analysis. FTY720 and DMS pretreatment significantly inhibited S1P‐induced fibrotic changes in the UUO mice (Fig. 6). In the interstitial injury score, the UUO mice exhibited increased the score in the left kidney compared with the control right kidney (from 0.83 ± 0.31 to 7.67 ± 0.49; Fig. 7), and FTY720 and DMS pretreatment significantly decreased the scores (FTY720: from 7.67 ± 0.49 to 3.0 ± 0.26, DMS: from 7.67 ± 0.49 to 3.33 ± 0.21; Fig. 7). In the immunohistochemical study, the UUO mice exhibited increased areas of staining in the left kidney compared with the control right kidney (fibronectin: from 2.34 ± 0.15 to 13.03 ± 0.28, COL1: from 1.52 ± 0.39 to 6.71 ± 3.39; Fig. 7), and FTY720 and DMS pretreatment significantly decreased the staining intensity of fibronectin and COL1 (fibronectin: after FTY720: from 13.03 ± 0.28 to 5.39 ± 1.27, after DMS: from 13.03 ± 0.28 to 6.04 ± 1.46; COL1 after FTY720: from 6.71 ± 3.39 to 1.88 ± 0.23, after DMS: from 6.71 ± 3.39 to 1.80 ± 0.19; Fig. 8). These results suggest that S1P induced renal fibrosis both in vitro and in vivo. Moreover, FTY720 and DMS inhibited renal fibrosis both in vitro and in vivo.

### Antifibrotic effects of FTY720 and DMS (UUO of nude mouse)

**Figure 9.**
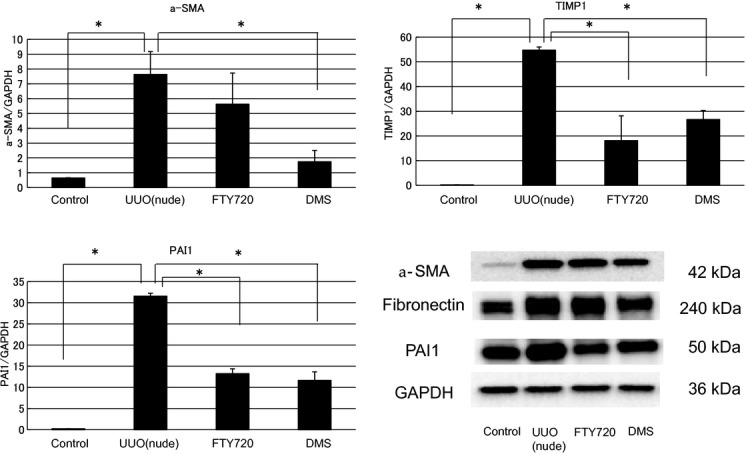
Examination of the antifibrotic effects of FTY720 and DMS in the UUO of nude mouse model by real‐time PCR and western blotting. In nude mice, the mRNA expression levels of the various fibrotic markers were significantly increased in the left kidney compared with the control right kidney. FTY720 and DMS pretreatment significantly inhibited the S1P‐induced fibrotic changes in the nude UUO mice. *N* = 3. UUO treatment of nude mice upregulated a‐SMA, fibronectin, and PAI1 expression levels. A representative western blot is shown. These effects on the protein levels were attenuated by FTY720 or DMS addition. Data are presented as means ± SE. *P < 0.05. UUO, unilateral ureteral obstruction; DMS, *N*,*N*‐dimethylsphingosine; a‐SMA, alpha‐smooth muscle actin; PAI1, plasminogen activator inhibitor‐1.

**Figure 10.**
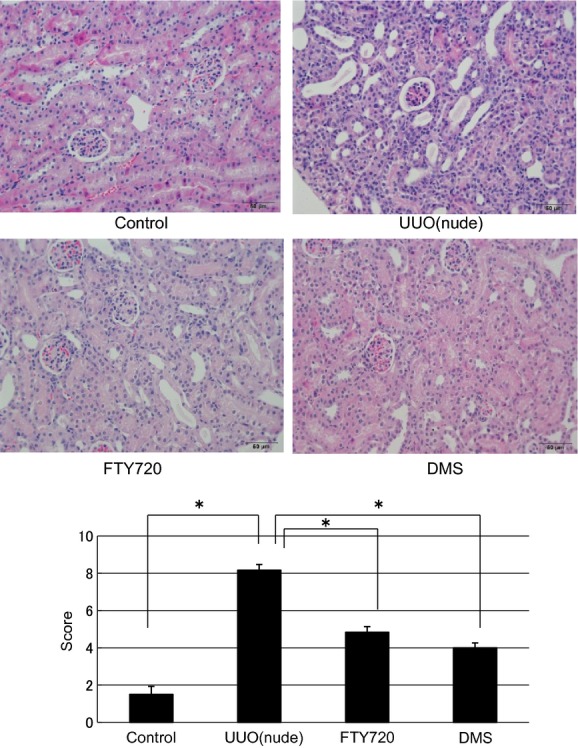
Examination of hematoxylin and eosin staining in the UUO of nude mouse model (magnification of 400×). In the interstitial injury score, the nude mice exhibited increased the score in the left kidney compared with the control right kidney (from 1.50 ± 0.43 to 8.17 ± 0.31), and FTY720 and DMS pretreatment significantly decreased the scores (FTY720: from 8.17 ± 0.31 to 4.83 ± 0.31, DMS: from 8.17 ± 0.31 to 4.0 ± 0.26). *N* = 6. Data are presented as means ± SE. *P < 0.05. UUO, unilateral ureteral obstruction; DMS, *N*,*N*‐dimethylsphingosine.

**Figure 11.**
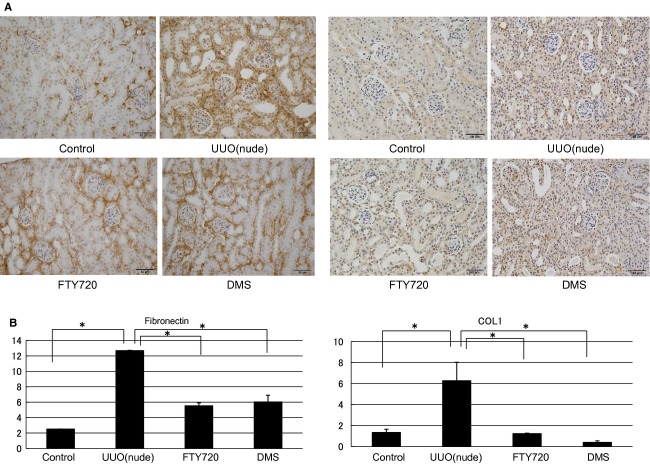
Examination of immunohistochemical staining in the UUO of nude mouse model. Immunohistochemistry showed that the stained area was expanded in the left kidney compared with the control right kidney in the nude mice. In the kidney of the nude UUO mouse model, FTY720 and DMS pretreatment significantly decreased the staining intensity of fibronectin and COL1. *N* = 3. Data are presented as means ± SE. *P < 0.05. UUO, unilateral ureteral obstruction; DMS, *N*,*N*‐dimethylsphingosine; COL1, collagen type 1.

In this investigation, nude mouse was used to examine the direct participation of S1P in fibrosis. In in vivo nude mice, the mRNA expression levels of various fibrotic markers in the left kidney were significantly increased compared with those in the control right kidney (a‐SMA/GAPDH ratio: from 0.65 ± 0.02 to 7.63 ± 1.54, TIMP1/GAPDH ratio: from 0.20 ± 0.04 to 54.73 ± 1.31, PAI1/GAPDH ratio: from 0.22 ± 0.01 to 31.58 ± 0.69; Fig. 9). FTY720 and DMS pretreatment significantly inhibited the S1P‐induced fibrotic changes at the gene level in the nude UUO mice (a‐SMA/GAPDH ratio: after FTY720: from 7.63 ± 1.54 to 5.62 ± 2.10, after DMS: from 7.63 ± 1.54 to 1.74 ± 0.77; TIMP1/GAPDH ratio: after FTY720: from 54.73 ± 1.31 to 18.12 ± 10.06, after DMS: from 54.73 ± 1.31 to 26.68 ± 3.67; PAI1/GAPDH ratio: after FTY720: from 31.58 ± 0.69 to 13.26 ± 1.11, after DMS: from 31.58 ± 0.69 to 11.65 ± 2.03; Fig. 9). Moreover, similar results were obtained with western blotting analysis (Fig. 9). In the interstitial injury score, the nude mice exhibited increased the score in the left kidney compared with the control right kidney (from 1.50 ± 0.43 to 8.17 ± 0.31; Fig. 10), and FTY720 and DMS pretreatment significantly decreased the scores (FTY720: from 8.17 ± 0.31 to 4.83 ± 0.31, DMS: from 8.17 ± 0.31 to 4.0 ± 0.26; Fig. 10). In the immunohistochemical study, expansion of the stained area was observed in the left kidney compared with the right control kidney (fibronectin: from 2.5 ± 0.00 to 12.67 ± 0.04; COL1: from 1.33 ± 0.32 to 6.27 ± 1.76; Fig. 11). In the kidney of the nude UUO mice, FTY720 and DMS pretreatment significantly decreased the staining intensity of fibronectin and COL1 (fibronectin: after FTY720: from 12.67 ± 0.04 to 5.53 ± 0.38, after DMS: from 12.67 ± 0.04 to 6.01 ± 0.88; COL1: after FTY720: from 6.27 ± 1.76 to 1.22 ± 0.05, after DMS: from 6.27 ± 1.76 to 0.39 ± 0.15; Fig. 11). These results suggest that S1P induced fibrosis not through an inflammatory pathway, but S1P had direct fibrotic effects.

**Figure 12.**
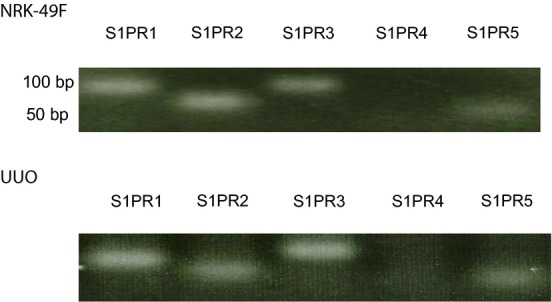
The expression of S1P receptor 1‐5 mRNA using RT‐PCR in NRK‐49F cells and UUO. In vitro and in vivo showed mRNA expressions of S1PR1, 2, 3, and 5, but not S1PR4. UUO, unilateral ureteral obstruction; S1P, sphingosine‐1‐phosphate; NRK‐49F, normal rat kidney interstitial fibroblast.

### The expression of S1P receptors 1‐5 mRNA using RT‐PCR in NRK‐49F cells and UUO

In vitro and in vivo showed mRNA expressions of S1P1, 2, 3, and 5, but not S1P4.

## Discussion

In each organ, fibrosis is a very important problem that is related to organ dysfunction. In this study, the relationship between renal fibrosis and S1P was investigated.

In the in vitro study, dose‐dependent increases in the levels of fibrotic markers were observed both at the gene and protein levels by stimulation with S1P. Attenuation of the S1P‐induced fibrotic markers was observed with exposure to FTY720 and DMS. These results suggest that S1P is strongly involved in fibrotic mediators. In addition, in this study, cell migration, a marker of fibrotic change, was increased by S1P compared with that in the control, and these changes were suppressed by FTY720 and DMS. These results also suggest that S1P is involved in the mechanisms of renal cell fibrosis. A previous study has shown that platelet‐derived growth factor stimulated fibrosis in hepatic stellate cells, and FTY720 counteracted these effects and suppressed the fibrotic effects and migration (Brunati et al. 2008). These findings were consistent with those of the present study.

UUO is a model used to examine fibrosis. With UUO, renal tubulointerstitial fibrosis results from ligating the left renal tubule. In this study, UUO‐induced renal tubulointerstitial fibrosis was suppressed at the gene and protein levels by FTY720 and DMS. Similar results were observed in the hematoxylin and eosin staining (H&E) and immunohistochemical studies. Taken together, the in vitro and in vivo findings of the effects of fibrosis suggested that S1P is involved in fibrosis. Renal fibrosis has been shown to occur through inflammatory pathways involving S1PR1 (Okusa and Lynch 2007). In this study, the direct effects of S1P on renal fibrosis and not those mediated by inflammation were examined in a nude mouse. Nude mice have defective thymi. Thus, their cell‐mediated immune responses are completely defective, and their humoral immunity is somewhat defective. Thymi are considered important in cell‐mediated immunity and particularly in the interstitial fibrosis of UUO. Therefore, the effects of immune cell‐mediated immunity can be excluded by UUO in nude mice. In this model that excluded the involvement of the inflammatory pathway in the S1P‐mediated effects in fibrosis, the direct effects of S1P in fibrosis could be investigated. Thus, the UUO model of nude mice used indicated that a significant difference in fibrosis was induced by S1P at the gene level, at the protein level, and in the H&E and immunohistochemical studies, and those changes were suppressed by FTY720 and DMS. These results suggest that the direct effects of S1P in fibrosis were not immune mediated. These effects of FTY720 and DMS suggest that they might be a treatment for CKD after the acute phase in the future. Athymic animals are T‐cell deficient, but have a full complement of other leukocyte populations, including macrophages. As S1P can regulate macrophage activity, estimation of infiltrating macrophages with or without S1P antagonism in both normal and nude mice will be considered in future.

S1P was once considered to just be a breakdown product of ceramide (Billich and Baumruker 2008). However, the fact that the function of S1P has been conserved from plants to higher organisms highlights its importance as a pivotal signaling molecule in normal functioning physiology and pathophysiology. S1P is a multipotent lipid mediator that acts on G‐protein‐coupled S1PR on the cell surface. S1P is produced by the phosphorylation of sphingosine by sphingosine kinases, and it acts as a ligand for specific receptors that are differentially expressed and that are coupled to various G proteins. These receptors regulate angiogenesis, vascular maturation, cardiac development, and immunity and are important for directed cell movement. In addition to the well‐established effects of S1P, several lines of evidence are accumulating that demonstrate that S1P is an important regulator of fibrosis. Previous studies have shown that S1P induces fibrosis in retinal pigmented epithelial cells (Swaney et al. 2008) and hepatocytes (Ikeda et al. 2009) and that S1P treatment increases a‐SMA expression in lung fibroblasts (Kawashima et al. 2012). There are also a report that S1P has both pro‐ and antifibrotic effects (Schwalm et al. 2013) and a report that sphingosine kinase‐1 activity has antifibrotic effects in a human podocyte cell line in the fibrotic process (Shuyu et al. 2009). S1P shows various effects by the situation in this way. These two reports are very important, and are also research on renal interstitial fibrosis important for exacerbate of kidney. This study demonstrated that S1P stimulates cell and tissue fibrotic processes in vitro and in vivo. Over the past several years, significant progress has been made in our understanding of the cellular and molecular mechanisms of renal fibrosis. The current model of renal fibrogenesis is similar to the model of wound‐healing response to injury (Watterson et al. 2007). After the initial injury, the affected kidney tissues undergo a series of events in an attempt to repair and recover from the damage. Compelling evidence has indicated that interstitial inflammation plays a central role in the loss of renal function in CKD. The combined effects of interstitial inflammation, oxidative stress, and local angiotensin II activity result in the disruption of glomerulus‐tubule continuity, the development of pathogenic hypoxia, the generation of myofibroblasts and interstitial fibrosis, and impairments in the protective autoregulation of glomerular blood flow that leads to glomerulosclerosis Mitobe et al. **(**2010). Recent evidence has shown that S1P acts on several types of target cells and is engaged in profibrotic inflammatory processes and fibrogenic processes through multiple mechanisms, which include vascular permeability changes, leukocyte infiltration and migration, proliferation, and myofibroblast differentiation (Cencetti et al. 2010). Many of these S1P actions are receptor subtype specific. In these actions, S1P has multiple cross reactions with other cytokines, particularly TGF‐*β* (Kono et al. 2007), which plays a major role in fibrosis.

Five subtypes of S1PRs have been identified. There have been reports of fibrosis and S1P in each cell in each organ, and differences in the effects of S1PRs have been reported in each organ. For example, S1PR3 is related to fibrosis in cardiac ventricular fibroblasts (Takuwa et al. 2010), S1PR2 is involved in a diabetic nephropathy model (Huang et al. 2012), and a relationship has been found between S1PR3 and fibrosis in myofibroblasts (Keller et al. 2007). In addition, there have been some reports that S1P and TGF‐*β* are associated in the lung, that a‐SMA is induced by TGF‐*β* that stimulates S1P, and that TGF‐*β* inhibits S1PR1 and stimulates S1PR3 in particular (Kawashima et al. 2012). An association between CTGF and S1PR2 has been reported in Wilms' tumor (Li et al. 2008). Unique tissue distribution of the receptor subtypes and the differing signaling pathways and downstream cellular effects resulting from S1PR subtype activation underscore the need to identify and test novel S1PR subtype‐specific compounds for the treatment of various disorders. Clinical trials have recently been conducted on the use of compound‐targeted S1PR for the development of agents for autoimmune diseases and renal transplantation. S1PR compounds are being used as clinical treatments for multiple sclerosis (Gasperini et al. 2013) and in preclinical studies for a number of different disorders. While FTY720 has been shown to activate S1PR at times (Chiba 2005), it has been shown to inhibit it at other times (Liu et al. 2013). Thus, although FTY720 is used as the agonist, there are reports in which FTY720 is an agonist functionally or an antagonist functionally. In this study, FTY720 acted as a functional antagonist of S1P. S1P and other bioactive lipids have been implicated in the regulation of wound healing and tissue repair (Watterson et al. 2007). Given the importance of the fibrotic system in kidney disease, S1PR compounds hold great promise for the treatment of various kidney disorders. FTY720 is an immunomodulator, but there have been reports of the antifibrotic effects of this compound in other tissues (Peters et al. 2004; Man et al. 2005). In this study, fibrotic changes were suppressed in NRK‐49F cells and in the UUO model in the presence of FTY720 and DMS. These results suggest that FTY720 and DMS may have beneficial effects against renal fibrosis. Application of the treatment of drug‐related phospholipids, such as FTY720 and DMS, is also expected in the future. We must elucidate the direct and indirect relationships between fibrotic factors, such as TGF‐*β* and CTGF, and S1P.

The biological properties and roles of S1P have been explored in the kidney. A number of reports have attempted to clarify the physiological and pathophysiological roles of S1P in the kidney. In the acute kidney injury model, namely, the renal ischemia reperfusion model (IRI), both innate and adaptive immunity play quite important roles in the establishment of the pathophysiology of acute tissue injury in the kidney (Yoshida et al. 2008). It has been speculated that S1P is implicated in the immune process of IRI. FTY720 has been shown to be effectively protective against experimental IRI (Awad et al. 2006). Furthermore, it has been clarified that the primary tissue protective role of S1P is mediated by the S1PR1 (Awad et al. 2006; Okusa and Lynch 2007), resulting in a decrease in immune‐mediated cells and inflammatory cells in the lesions of the kidney. In addition, the role of S1P in a chronic model of kidney disease has been explored in a rat model of anti‐Thy1‐induced chronic progressive glomerulosclerosis (Peters et al. 2004). These findings suggest that FTY720 has beneficial effects and is able to reduce the progression of the disease toward chronic tubulointerstitial fibrosis and renal insufficiency through its ability to deplete lymphocytes and stop the inflammatory reactions (Okusa and Lynch 2007; Peters et al. 2004). In this study, an examination of the relationship between S1P and fibrosis was applied to a UUO model and a UUO model of nude mice, and FTY720 was shown to have direct beneficial effects on fibrosis. In the future, further investigations and clinical applications are required to learn more about the best ways to prevent fibrosis.

In conclusion, this study results showed that S1P is a hitherto unrecognized profibrotic mediator in the renal tubulointerstitium that functions through S1PR signaling pathways and that, in part, has direct fibrotic effects. Moreover, FTY720 and DMS could have effects in addition to their immunosuppressive effects in the antifibrotic activity.

## Acknowledgments

We particularly wish to thank Ai Munekawa, Mayuko Futaya, and Atsuko Teraoka for their excellent technical assistance.

## Conflicts of Interest

None declared.
